# What may an adrenal mass conceal? Adrenal gastrointestinal stromal tumor: a case report

**DOI:** 10.1093/jscr/rjae229

**Published:** 2024-04-10

**Authors:** Mahmoud Alafifi, Abderrahmane Doumer, El Mostaoha Abdi, Mehdi Safieddine, Amine Moataz, Mohamed Dakir, Adil Debbagh, Rachid Aboutaieb

**Affiliations:** Department of Urology, University Hospital Center IbnRochd Casablanca, 19 Tarik ibnou quartiers des hôpitaux, Casablanca 9154, Morocco; Faculty of Medicine and Pharmacy of Casablanca, 19 Tarik ibnou quartiers des hôpitaux, Casablanca 9154, Morocco; Department of Urology, University Hospital Center IbnRochd Casablanca, 19 Tarik ibnou quartiers des hôpitaux, Casablanca 9154, Morocco; Faculty of Medicine and Pharmacy of Casablanca, 19 Tarik ibnou quartiers des hôpitaux, Casablanca 9154, Morocco; Department of Urology, University Hospital Center IbnRochd Casablanca, 19 Tarik ibnou quartiers des hôpitaux, Casablanca 9154, Morocco; Faculty of Medicine and Pharmacy of Casablanca, 19 Tarik ibnou quartiers des hôpitaux, Casablanca 9154, Morocco; Department of Urology, University Hospital Center IbnRochd Casablanca, 19 Tarik ibnou quartiers des hôpitaux, Casablanca 9154, Morocco; Faculty of Medicine and Pharmacy of Casablanca, 19 Tarik ibnou quartiers des hôpitaux, Casablanca 9154, Morocco; Department of Urology, University Hospital Center IbnRochd Casablanca, 19 Tarik ibnou quartiers des hôpitaux, Casablanca 9154, Morocco; Faculty of Medicine and Pharmacy of Casablanca, 19 Tarik ibnou quartiers des hôpitaux, Casablanca 9154, Morocco; Department of Urology, University Hospital Center IbnRochd Casablanca, 19 Tarik ibnou quartiers des hôpitaux, Casablanca 9154, Morocco; Faculty of Medicine and Pharmacy of Casablanca, 19 Tarik ibnou quartiers des hôpitaux, Casablanca 9154, Morocco; Department of Urology, University Hospital Center IbnRochd Casablanca, 19 Tarik ibnou quartiers des hôpitaux, Casablanca 9154, Morocco; Faculty of Medicine and Pharmacy of Casablanca, 19 Tarik ibnou quartiers des hôpitaux, Casablanca 9154, Morocco; Department of Urology, University Hospital Center IbnRochd Casablanca, 19 Tarik ibnou quartiers des hôpitaux, Casablanca 9154, Morocco; Faculty of Medicine and Pharmacy of Casablanca, 19 Tarik ibnou quartiers des hôpitaux, Casablanca 9154, Morocco

**Keywords:** adrenal tumor, extragastrointestinal stromal tumors

## Abstract

Extraintestinal gastrointestinal stromal tumors (GISTs) are extremely rare, and adrenal GISTs are even exceptional. Only three cases have been reported in the literature thus far, the current case being the fourth. This case demonstrates the need of including extraintestinal GIST in the differential diagnosis when investigating adrenal tumors. Herein, we present a case of adrenal GIST diagnosed in a 60-year-old female patient who had a left adrenal GIST surgically removed as an adrenal tumor.

## Introduction

The discovery of an adrenal mass is a common occurrence nowadays. While adrenal tumors can cause a range of symptoms, they are most typically discovered incidentally during imaging examinations such as computed tomography (CT) scans or magnetic resonance imaging (MRI) that are being conducted for another reason.

Imaging advancements, as well as the development of rigorous histological examinations of these masses, have allowed for a more accurate diagnosis.

Nevertheless, various main adrenal, periadrenal, and secondary tumors, as well as pseudoadrenal and infectious masses, are included in the differential diagnosis of adrenal tumors. Apart from myelolipomas, mesenchymal adrenal tumors including liposarcomas, angiosarcomas, desmoid, and myelofibroblastic tumors, among others, are an extremely uncommon neoplasm, accounting for <1% of all adrenal tumors in surgical and pathological series [1].

Extragastrointestinal stromal tumors (EGISTs) are tumors that have the same histological and immunohistochemical properties as tubular GISTs but do not have a connection to the tubular gastrointestinal tract [1].

EGISTs emerging from the adrenal gland are exceedingly rare; till now, only three cases have been reported in the literature.

Herein, we present a case of adrenal GIST diagnosed in a 60-year-old female patient who had a left adrenal GIST surgically removed as an adrenal tumor.

## Case report

A 60-year-old woman, with a history of cholecystectomy 35 years ago, presented for lumbar heaviness with left low back pain with no other associated signs, evolving in a context of deteriorating general condition.

On admission, the vital signs were stable. She had left lumbar tenderness, and negative lumbar contact.

On biological assessment, all the parameters were normal, with a normal plasma level of metanephrines/normetanephrines.

**Figure 1:**
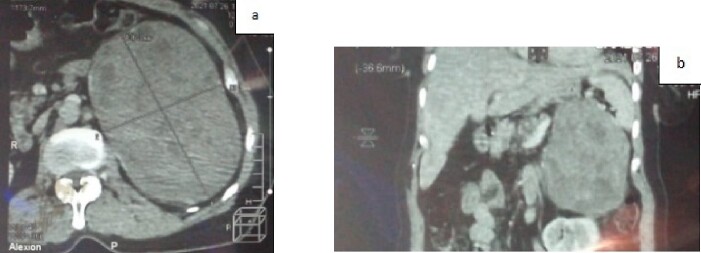
a. CT scan of the abdomen showing a voluminous left adrenal mass (axial section); b. CT scan of the abdomen showing a voluminous left adrenal mass (coronal section).

**Figure 2:**
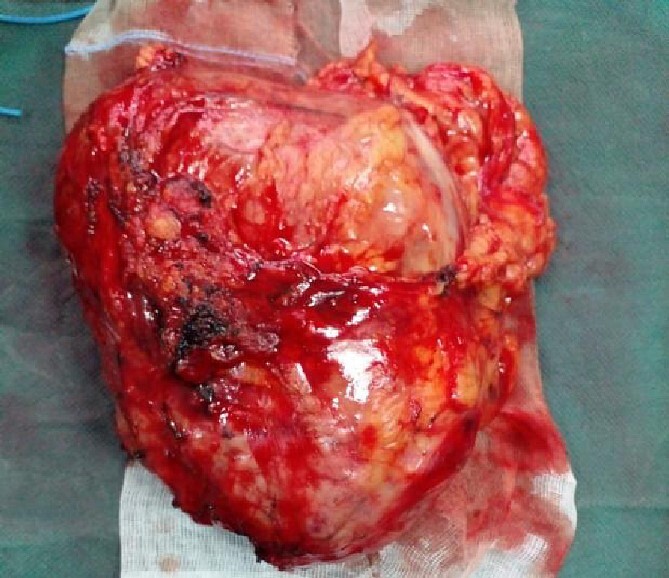
Operation specimen of a left adrenal mass.

An abdominal CT scan confirmed the presence of a voluminous left adrenal mass (Fig. 1). After further discussion at the multidisciplinary meeting, surgical resection was performed, with excision of an 18 cm left adrenal mass (Fig. 2).

The patient was discharged on Day 4 following the surgical resection without complication. She was reviewed 2 weeks postoperatively to discuss pathology results; 4 months following surgical resection the patient is well.

**Figure 3:**
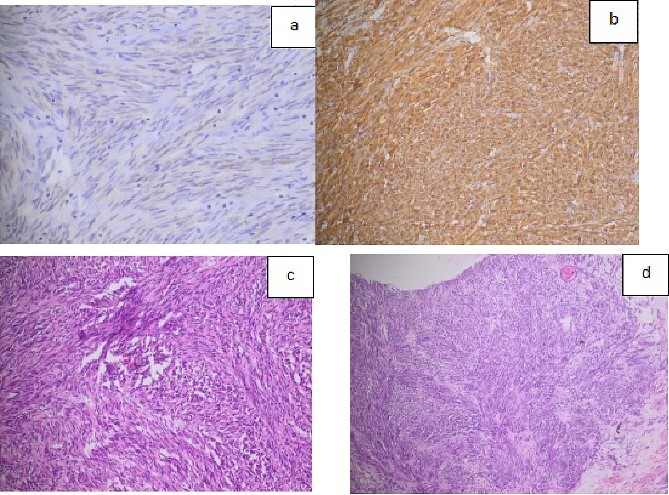
a. Histological image showing the expression of C-kit or CD117 (almost constant in GISTs); b. Histological image showing the expression of CD34 marker; c. Histological expression of DOG 1 marker; d. microscopic image of HE^*^200 expression.

Histology showed low nuclear grade spindle cell tumor proliferation. The immunohistochemical study was in favor of a GIST (Fig. 3).

## Discussion

GISTs are the most prevalent mesenchymal tumors of the gastrointestinal system, with the stomach (about 60%) and small intestine (about 30%) being the most common sites of development. EGISTs are mesenchymal tumors that have immunohistochemical markers with GISTs but do not have a tubular gastrointestinal tract connection [1].

GISTs often manifest between the fifth and eighth decades of life. The majority of individuals are diagnosed between the ages of 60 and 70. They affect both sexes equally, with a little male predominance. Although GISTs are thought to affect 3000–4000 people each year, the true incidence of these neoplasms is unknown due to the large number of asymptomatic patients who go undiagnosed [1].

GISTs are thought to be derived from interstitial Cajal (ICC) (pacemaker cells) found in the muscular layer of the gastrointestinal walls. CD117 (c-kit, a transmembrane tyrosine kinase receptor) and CD34 (a highly glycosylated cell surface antigen) are found on ICCs. The immunohistochemical demonstration of CD117 and/or CD34 is a hallmark of GIST pathology [2].

Around 70% of patients are symptomatic, while the remainder have been asymptomatic for years, and their tumors are usually discovered by chance or during autopsy. Symptomatic patients typically have big tumors, and their symptoms are connected to their anatomical site and/or mass effect.

Abdominal pain, GI bleeding (including melena, hematemesis, and anemia), palpable mass, lethargy, bloating, early satiety, malaise, and weight loss are the most prevalent symptoms reported by patients. Although rare, tumor rupture and hemorrhaging can occur with these tumors, resulting in poor outcomes [1, 3].

On a CT scan, GISTs appear as lesions that run parallel to the stomach wall. They are diverse, with centrally placed changes and peripheral enhancement of undamaged sections. The solid region of an MRI of a large-sized GIST reveals a T1-weighted hyposignal and a T2-weighted hypersignal with substantial, usually peripheral, enhancement. The modified zones’ appearance varies. Malignant tumors with stomach invasion and desmoid tumor are the most common differential diagnosis. 18-FDG PET is crucial in determining the malignancy of extra-adrenal tumors [4].

Because two-thirds of EGIST are cancerous, surgical resection with a safe margin is usually advised. Having said that, surgery can differ based on the type of tumor. While laparoscopy is frequently used, open surgery is still an option if the tumor is sarcoma, >6 cm, or requires vascular control. Tyrosine kinase inhibitors have revolutionized the treatment of GISTs, especially unresectable tumors [4, 5]. In general, radiation and chemotherapy are unsuccessful against these malignancies [1].

GISTs often present as well-circumscribed gray to white exophytic masses originating from the wall in an anatomopathological examination. The tumor is frequently surrounded by a pseudocapsule, which comprises areas of bleeding, necrosis, and/or cystic degeneration. They exhibit either a spindle-cell or an epithelioid pattern under the microscope. The most common histological pattern in GIST cases (70%) is spindle cell. Sclerosing, hypercellular, palisading-vacuolated, and sarcomatous are some of the subtypes [1].

## Conclusion

EGISTs are extremely rare, and adrenal GISTs are even exceptional. Only three cases have been reported in the literature thus far, the current case being the fourth. This case demonstrates the need of including extraintestinal GIST in the differential diagnosis when investigating adrenal tumors. This unusual entity must be identified since it may provide a clinical and histological diagnostic issue. In such cases, an immunohistochemistry panel that includes CD117 and DOG1 is required to provide a definitive diagnosis.
